# Observation of σ-pore currents in mutant *h*Kv1.2_V370C potassium channels

**DOI:** 10.1371/journal.pone.0176078

**Published:** 2017-04-20

**Authors:** Pavel Tyutyaev, Stephan Grissmer

**Affiliations:** Institute of Applied Physiology, Ulm University,Ulm, Germany; Dalhousie University, CANADA

## Abstract

Current through the σ-pore was first detected in *h*Kv1.3_V388C channels, where the V388C mutation in *h*Kv1.3 channels opened a new pathway (σ-pore) behind the central α-pore. Typical for this mutant channel was inward current at potentials more negative than -100 mV when the central α-pore was closed. The α-pore blockers such as TEA^+^ and peptide toxins (CTX, MTX) could not reduce current through the σ-pore of *h*Kv1.3_V388C channels. This new pathway would proceed in parallel to the α-pore in the S6-S6 interface gap. To see whether this phenomenon is restricted to *h*Kv1.3 channels we mutated *h*Kv1.2 at the homologue position (*h*Kv1.2_V370C). By overexpression of *h*Kv1.2_V370C mutant channels in COS-7 cells we could show typical σ-currents. The electrophysiological properties of the σ-pore in *h*Kv1.3_V388C and *h*Kv1.2_V370C mutant channels were similar. The σ-pore of *h*Kv1.2_V370C channels was most permeable to Na^+^ and Li^+^ whereas Cl^-^ and protons did not influence current through the σ-pore. Tetraethylammonium (TEA^+^), charybdotoxin (CTX) and maurotoxin (MTX), known α-pore blockers, could not reduce current through the σ-pore of *h*Kv1.2_V370C channels. Taken together we conclude that the observation of σ-pore currents is not restricted to Kv1.3 potassium channels but can also be observed in a closely related potassium channel. This finding could have implications in the treatment of different ion channel diseases linked to mutations of the respective channels in regions close to homologue position investigated by us.

## Introduction

Earlier studies showed that mutation in voltage-gated and potassium channels could open other pathways besides the central α-pore through the complex channel molecules. These pathways could be described as alternative pores and were initially observed with mutations in the voltage-sensing domain (S1-S4) of the channels. For example by exchanging a positively charged arginine at position 362 in R1 S4 of the *Shaker* channel by cysteine or serine, an alternative pore (ω-pore) could be opened [1]. The ω-pore produce leak current conducting monovalent cations and is most permeable to K^+^. In addition, alternatives ω-pores in sodium channels could be observed with mutations in the voltage sensor S4 of Nav1.2 and Nav1.7 [2,3].

Kv1.2 and Kv1.3 channels are voltage-activated channels that open with depolarizations. Both channel proteins consist of four subunits. The N- and C-terminal regions of the channels are located at the intracellular side [4]. Each subunit of these channels contain six membrane-spanning regions (S1-S6) with a P-region between S5 and S6. This S5-P-S6 forms, together with three other subunits, the central, potassium selective α-pore. Segments S1-S4 form the voltage-sensing domain (VSD). This VSD controls the gates and is located around the pore domain [1,5]. Mutations in the VSD of the voltage-gated sodium channel Nav1.2 or the voltage-gated *Shaker* potassium channel can open another ion permeation pathway through the channel molecule [1–4, 6–7]. This new pathway through the VSD was described as ω-current, was selective for monovalent cations and was open at potentials when the α-pore was closed [1].

Yet another pathway, the σ-pore through a mutant potassium channel was described [8] in a valine to cysteine mutant channel at position 388 in *h*Kv1.3 (*Shaker* position 438, for a sequence alignment please see Table 1). This mutant *h*Kv1.3_V388C channel showed an additional inward current at membrane potentials more negative than -100 mV. This σ-current showed similarities to the ω-current that flows through the voltage-sensing domain of the R1C/S mutated *Shaker* channel described above: First, ω- and σ-currents can only be observed at potentials more negative than -100 mV, a potential range where the central α-pore is normally closed; second, ω- and σ-currents can be carried by different monovalent cations like Li^+^ and Cs^+^; third, extracellularly applied α-pore blockers reduced current through the α-pore, however, had no effect on the ω- or σ-current. Since the ω-current was carried best by K^+^ and the σ-current carried best by Na^+^, the authors concluded that the pathway of the ω-current was distinct from the pathway of the σ-current. Moreover, the *h*Kv1.3_V388C mutant channel not only showed a sustained current at potentials more negative than -100 mV in external solutions containing in mM [160 Na^+^ + 4.5 K^+^]_o_ but displayed normal current behavior in [164.5 K^+^]_o_ compared with the *h*Kv1.3_wt channel. Based on this normal current behavior in the *h*Kv1.3_V388C mutant channel in high potassium outside the authors concluded that the V388C mutation in *h*Kv1.3 generated a channel with two ion-conducting pathways. One, the central α-pore allowing K^+^ permeation in the presence of extracellular K^+^ and another pathway, the σ-pore, functionally similar but physically distinct from the ω-pathway.

**Table 1:** Sequence alignment of the different channels. The highlighted amino acid was mutated in *h*Kv1.2 and *h*Kv1.3 and correspond to position 438 in *Shaker*, 370 in *h*Kv1.2, 388 in *h*Kv1.3 and 71 in *KcsA*.

linker S5/P →	←P-region→	← linker P/S6
*Shaker*	…FFKSIPDAFWWAV**V**TMTTVGYGDMYPVGFWGKIVG…	459
*h*Kv1.2	…GFNSIPDAFWWAV**V**SMTTVGYGDMVPTTIGGKIVG…	391
*h*Kv1.3	…GFSSIPDAFWWAV**V**TMTTVGYGDMHPVTIGGKIVG…	409
*KcsA*	…QLITYRRALWWSV**E**TATTVGYGDLYPVTLWGRLVA…	92

According to the model of the mutant *h*Kv1.3_V388C channel, the exchange of the valine by cysteine, removing the two methyl groups of the valine at position 388 enlarged the space in between Y395 and W384 and may now allow the passage of ions [8]. The σ-pore was located behind the central α-pore at the back of the selectivity filter and proceeded parallel to the central α-pore. The entry of the σ-pore was located between the Tyr-395 of the GYG motif and the Trp-384 of the pore helix [8].

To find out whether the σ-pore is restricted to *h*Kv1.3 channels we mutated *h*Kv1.2, a very closely related voltage-gated potassium channel, at the homologue position (*h*Kv1.2_V370C) and observed current behavior identical to current through the σ-pore.

## Material and methods

### Molecular cloning and site directed mutagenesis

The *h*Kv1.2_wt template cDNA was a generous gift from Prof. Dr. O. Pongs (Institute for Neural Signal Processing, Center for Molecular Neurobiology, Hamburg Germany) and was cloned in the pRc/CMV vector (Invitrogen) and the mutagenesis was exactly performed according to the QuickChange TM mutagenesis protocol (Stratagene). The new *h*Kv1.2_V370C plasmid was sequenced and amplified in E. *coli*.

### Cell culture

For the expression of channels the adherent cell line COS-7 (passage 6 and 12, DSMZ no. ACC 60, Braunschweig, Germany) was used. The COS-7 cells were grown according to the standard protocol in DMEM high glucose with 10% FBS. The cells were incubated at 37°C, 5% CO_2_ with saturated humidity. Cells were grown to 95% confluence and transfected with 2 μg of total *h*Kv1.2_V370C DNA plus 0.2 μg of pEGFP-C1 (CLONTECH) DNA using FuGENE 6 (Roche Molecular Biochemicals). The cells were replated the day after transfection on poly-L-lysine-coated coverslips, and EGFP-positive cells were patch clamped 36–48 h after transfection, as described below.

### Electrophysiology

The patch-clamp measurements were performed as described earlier [8]. Briefly, measurements were performed at room temperature 19-22°C in the whole-cell configuration [9–10]. Cells were visualized with an inverted microscope Axiovert 25 (Carl Zeiss AG, Jena, Germany) installed on a vibration-isolation table (Newport Corporation, Irvine, USA) equipped with a xenon lamp and fluorescence detection unit. The amplifier EPC-9 (HEKA Elektronik GmbH, Lambrecht, Germany) was connected to a Dell computer running Patchmaster 2.0 data acquisition software. Currents were filtered through a 2.9 kHz Bessel Filter and capacitative and leakage currents were not subtracted. All voltage ramp protocols were preceded by a 100-ms prepulse to the starting potential to avoid complications associated with the slow “activation” of the σ-current. The analysis of the data was performed with the programs Fitmaster v2.15 (HEKA Elektronik GmbH) and Igor Pro 3.1.2 (Wave Metrics Inc., Lake Oswego, Oregon).

### Solution and chemicals

The measurements were performed in different external bath solutions. The composition of the external bath solutions used in the present study:[Na^+^]_o_: 160 mM NaCl, 4.5 mM KCl, 2 mM CaCl_2_, 1 mM MgCl_2_, 5 mM HEPES pH 7.4 adjusted with NaOH; [X^+^]_o_: 164.5 mM XCl, 2 mM CaCl_2_, 1 mM MgCl_2_, 5 mM HEPES adjusted pH 7.4 with XOH, X stands for K^+^, Rb^+^, Cs^+^, Li^+^ and NH_4_^+^. Osmolarity of the bath solutions was 300-310 mOsm. The internal pipette solution contained 145 mM KF, 2 mM MgCl_2_, 10 mM HEPES, 10 mM EGTA and was adjusted with KOH to pH 7.2 and the osmolarity was 310 mOsm. Charybdotoxin, CTX (Bachem, Bubendorf, Switzerland) and maurotoxin, MTX (Sigma-Aldrich, Saint Louis, USA) were dissolved in bath solution with 0.1% BSA.

### Modeling

The model of the σ-pore in *h*Kv1.2_V370C was created as described earlier for the *h*Kv1.3_V388C mutant channel [8]. Briefly, we mutated V370C in the *h*Kv1.2 wt (2A79) monomer with the help of the Deep Viewer software (Swiss PDB viewer, Expasy Server) followed by the creation of the *h*Kv1.2_V370C homotetramer. The σ-pore was simulated with CAVER software (Loschmidt Laboratories, http://www.caver.cz) and visualized with PyMOL viewer (DeLano Scientific LLC, Schrödinger).

## Results and discussion

The single point mutation V370C (*Shaker* position 438) in the *h*Kv1.2 background channel showed an inward current at potentials more negative than -100 mV similar to the σ-current found in the homologous *h*Kv1.3_V388C mutant channel [8] and different from the ω-current [1,6,7]. Below we characterized the electrophysiological and pharmacological properties of the inward current in *h*Kv1.2_V370C mutant channels and compared it with the known properties of the σ-current found in the *h*Kv1.3_V388C mutant channels.

### The substitution of valine 370 with cysteine in *h*Kv1.2 displays an inward current similar to the σ-current of *h*Kv1.3_V388C channels

**Fig 1:**
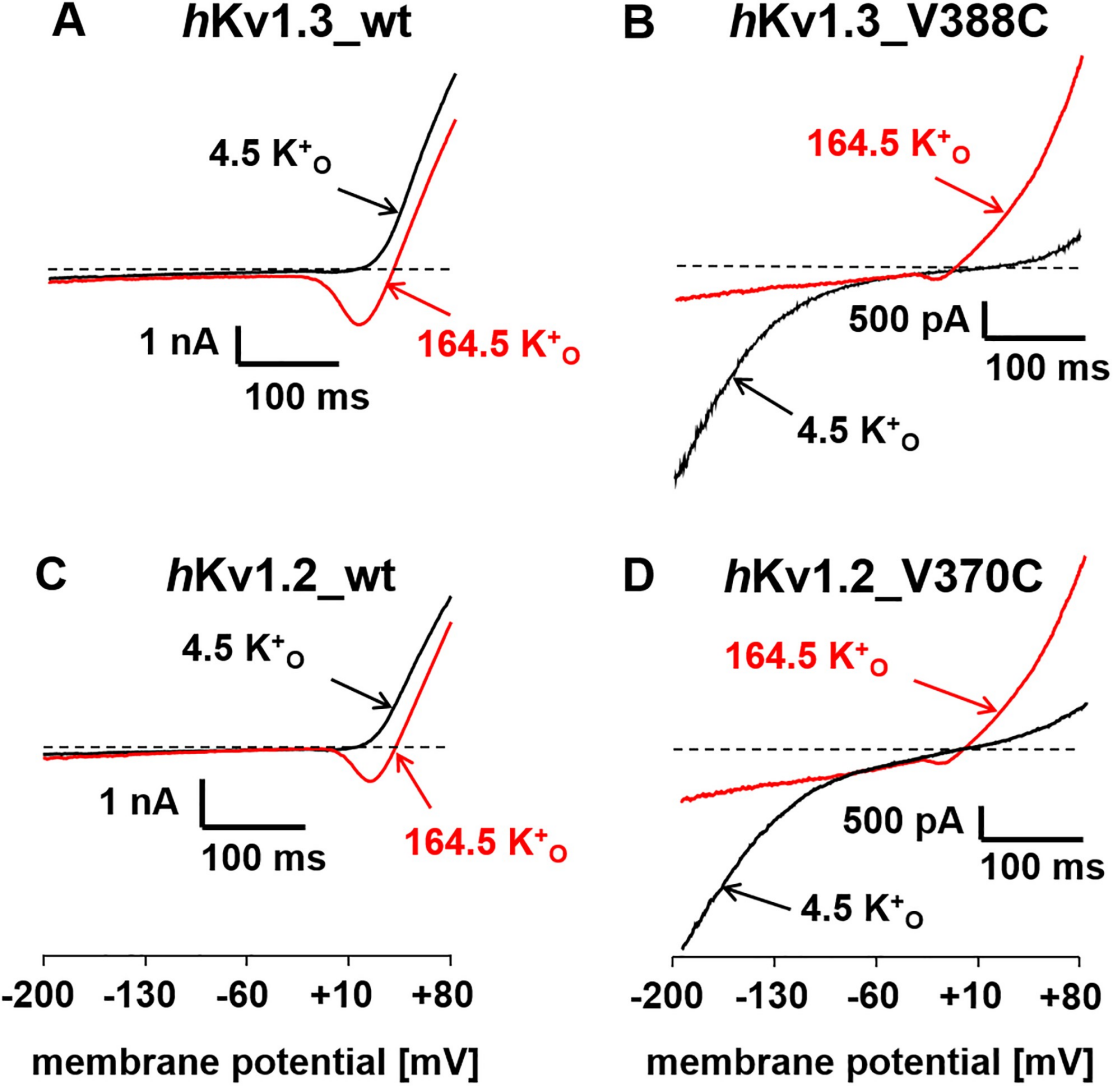
*h*Kv1.2_V370C mutant channels promote an inward current at potentials more negative than -100 mV, similar to *h*Kv1.3_V388C mutant channels. Ramp currents through *h*Kv1.3_wt (*A*), *h*Kv1.3_V388C (*B*), *h*Kv1.2_wt (*C*) and *h*Kv1.2_V370C (*D*) mutant channels in [160 Na^+^ + 4.5 K]_o_ (black traces) and [164.5 K^+^]_o_ (red traces) external bath solution. The currents were elicited by 400-ms voltage ramps from -200 to +80 mV every 30 s from a holding potential of -80 mV.

Fig 1 shows in the top row (*A*,*B*) typical ramp currents through *h*Kv1.3_wt (*A*) and *h*Kv1.3_V388C (*B*) mutant channels in bathing solutions containing either 4.5 (black traces) or 164.5 mM [K^+^]_o_ (red traces). As described earlier [8] a large inward current at potentials more negative than -60 mV can only be observed through the *h*Kv1.3_V388C mutant channels in a bathing solution containing 4.5 mM [K^+^]_o_ (*B*, black trace) and not through the *h*Kv1.3_wt channels or in a bathing solution containing 164.5 mM [K^+^]_o_. This inward current had been demonstrated to be due to current flowing through the σ-pore [8]. An almost identical current behavior is shown in the bottom row (*C*,*D*) of Fig 1, where ramp currents through *h*Kv1.2_wt (*C*) and *h*Kv1.2_V370C (*D*) mutant channels are shown in bathing solutions as described for Fig 1*A* and 1*B*. It seems that the V370C mutation in *h*Kv1.2, that is homologue to the *h*Kv1.3_V388C mutation, can also create an inward current at potentials more negative than -60 mV in a bathing solution containing 4.5 mM [K^+^]_o_ indicating to us the presence of the σ-pore pathway in the *h*Kv1.2_V370C mutant channels.

**Fig 2:**
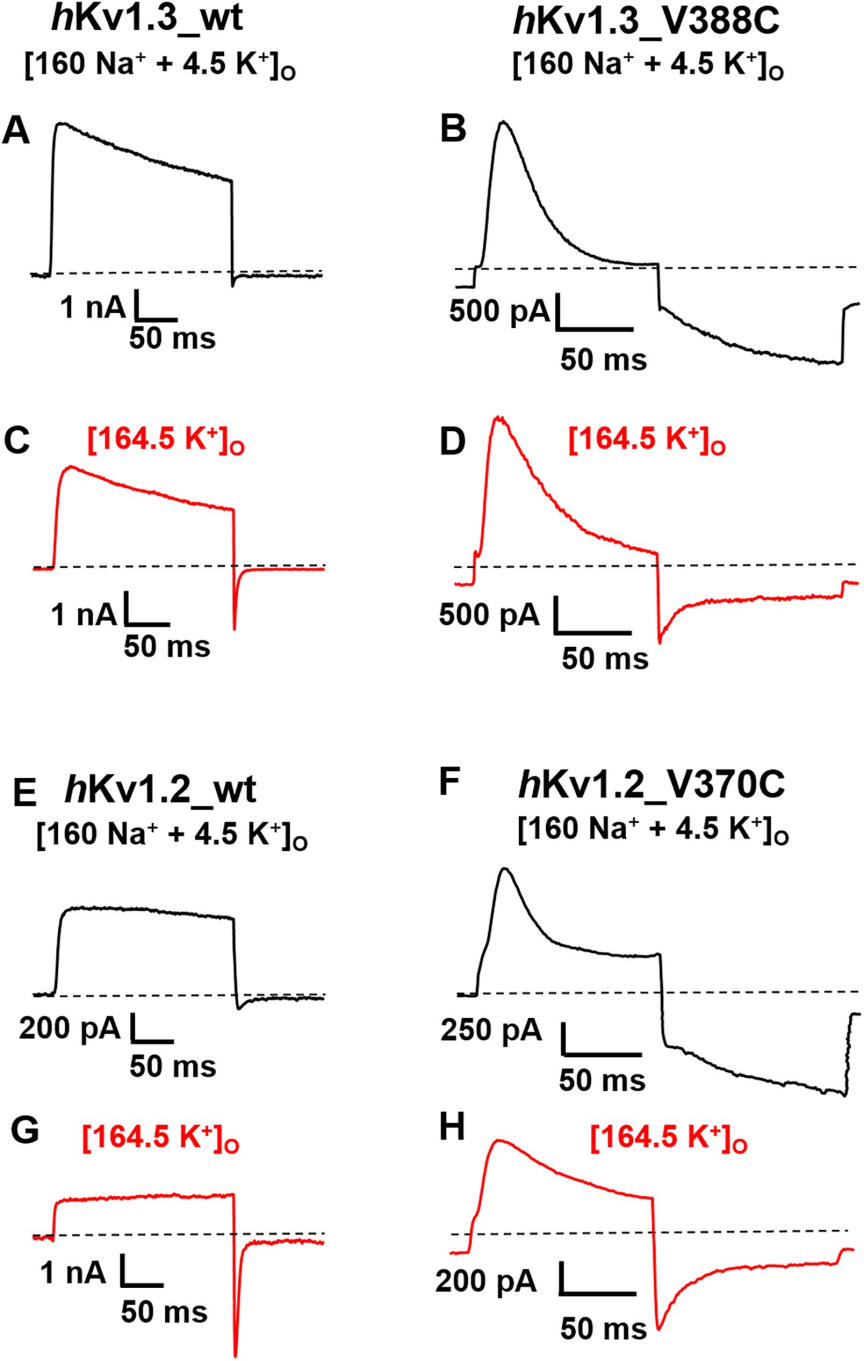
Similarity of inward current in *h*Kv1.2_V370C and *h*Kv1.3_V388C mutant channels. Currents through wt *h*Kv1.3 (*A*,*C*), wt *h*Kv1.2 (*E*,*G*), *h*Kv1.3_V388C (*B*,*D*) and *h*Kv1.2_V370C (*F*,*H*) channels generated with 100-ms (*B*,*D*,*F*,*H*) or 200-ms (*A*,*C*,*E*,*G*) depolarizing pulses from the holding potential of -120 mV to +40 mV followed by 100-ms hyperpolarizing pulses to -180 mV in [160 Na^+^ + 4.5 K^+^]_o_ (black traces) and in [164.5 K^+^]_o_ (red traces) external bath solution.

To confirm this assumption we performed the experiments shown in Fig 2. In the *h*Kv1.3_V388C mutant channel in [160 Na^+^ + 4.5 K^+^]_o_ (Fig 2*B*) we observed an outward current at +40 mV through the α-pore that inactivated much faster than the wild type (Fig 2*B*) together with an inward current at -180 mV. In comparison, the *h*Kv1.3 mutant channel in [164.5 K^+^]_o_ (Fig 2*D*) showed slightly slower inactivation at +40 mV compared with that in [160 Na^+^ + 4.5 K^+^]_o_ (Fig 2*B*). At -180 mV in [164.5 K^+^]_o_ we could observe a current that deactivated slower compared with *h*Kv1.3_wt (Fig 2*C*), however, with a smaller sustained inward current as seen in [160 Na^+^ + 4.5 K^+^]_o_. These observations are in agreement with earlier findings [8]. Similar observations regarding current through the α- and σ-pore as described above for *h*Kv1.3_V388C mutant channels in normal and high external potassium solutions can be made for currents through *h*Kv1.2_V370C mutant channels: At +40 mV in [160 Na^+^ + 4.5 K^+^]_o_ an outward current through the α-pore of the *h*Kv1.2_V370C mutant channels (Fig 2*F*) can be seen that inactivated much faster than in the wild type *h*Kv1.2 channel (Fig 2*E*) together with an inward current at -180 mV that increased during the 100-ms hyperpolarization in most of our experiments (21 out of 24) using this protocol. In a minority of these experiments (3 out of 24) the increase in σ-current amplitude at -180 mV was followed by a slight decrease during this 100-ms hyperpolarization. In [164.5 K^+^]_o_ the current at -180 mV deactivated slower (Fig 2*H*) compared with *h*Kv1.2_wt (Fig 2*G*) with a smaller sustained inward current compared to Fig 2*F*.

### σ-currents were not inhibited by CTX and MTX, known α-pore-blocking peptide toxins acting at the external mouth of the channel

**Fig 3:**
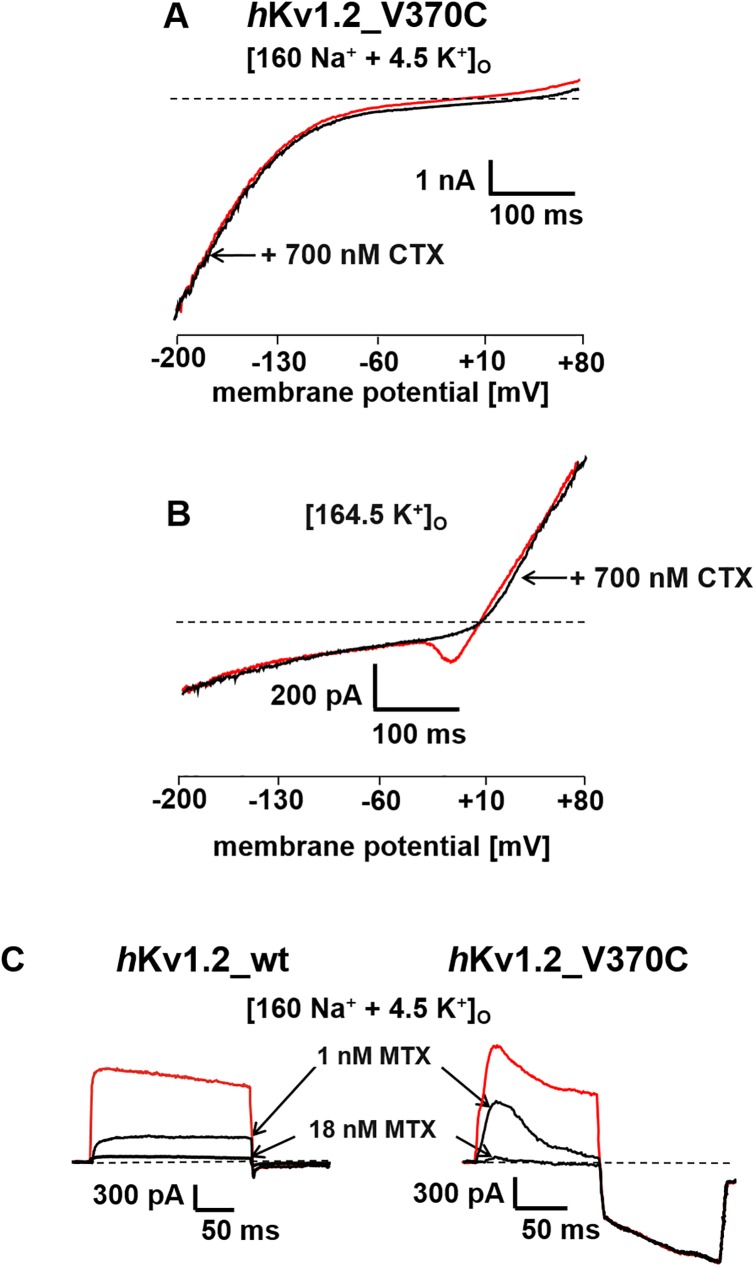
Effect of CTX and MTX on σ-current in the *h*Kv1.2_V370C mutant channel. *A* and *B* ramp currents through the *h*Kv1.2_V370C mutant channels in [160 Na^+^ + 4.5 K^+^]_o_ (*A*) and in [164.5 K^+^]_o_ (*B*) bath solution before and after extracellular application of CTX. Ramp currents were elicited as described in the legend to Fig 1. (*C*), effect of 1 and 18 nM MTX on currents through the α-pore of *h*Kv1.2_wt channels (*left*) and through the α- and σ-pores of *h*Kv1.2_V370C mutant channels in [160 Na^+^ + 4.5 K^+^]_o_, elicited with 100-ms depolarizing pulses from the holding potential of -120 mV to +40 mV followed by a 100-ms hyperpolarizing pulse to -180 mV every 30 s.

Fig 3*A* clearly shows that application of 700 nM CTX in a bathing solution containing 4.5 mM [K^+^]_o_ cannot block current through *h*Kv1.2_V370C mutant channels at potentials more negative than –60 mV indicating that CTX is unable to block current through the σ-pore while still able to block current through the central α-pore as can be seen in Fig 3*B* in a bathing solution containing 164.5 mM [K^+^]_o_ where the inward current dip in the potential range between -50 and 0 mV was completely abolished. At first glance one could wonder why CTX was unable to reduce outward current in this record. The answer to this phenomenon is similar to what has been reported earlier [8]: the time course of inactivation of the mutant *h*Kv1.2_V370C channel, even in high external potassium, shown in Fig 2*H*, is so fast that during the first 300 ms of the 400-ms voltage ramp (showing the inward current) the channel did completely inactivate. Therefore, the outward current in the ramp current shown in Fig 3*B* cannot go through the mutant *h*Kv1.2_V370C channel. We conclude that the outward current in Fig 3*B* is either a nonspecific leak current or flows through some other endogenous channels in the cell, for example through chloride channels.

In additional experiments we compared the application of 1 and 18 nM MTX on currents through *h*Kv1.2_wt (Fig 3*C*, *left*) and *h*Kv1.2_V370C mutant channels (Fig 3*C*, *right*). Through both channels the outward current through the α-pore at a potential of +40 mV during depolarization was similarly reduced. For example in the wild type *h*Kv1.2 channel 1 nM MTX reduced current to about one third of the control current and in the *h*Kv1.2_V370C mutant channel the same concentration reduced peak current to about one half. These current reductions indicate minor changes in the ability of MTX to block current through the α-pore of *h*Kv1.2_wt and *h*Kv1.2_V370C mutant channels. More importantly, amplitude and kinetic properties of the inward current through the σ-pore of the *h*Kv1.2_V370C mutant channel at -180 mV did not change (Fig 3*C*, *right*) at any of the applied MTX concentrations.

### Ion selectivity of the σ-current

**Fig 4:**
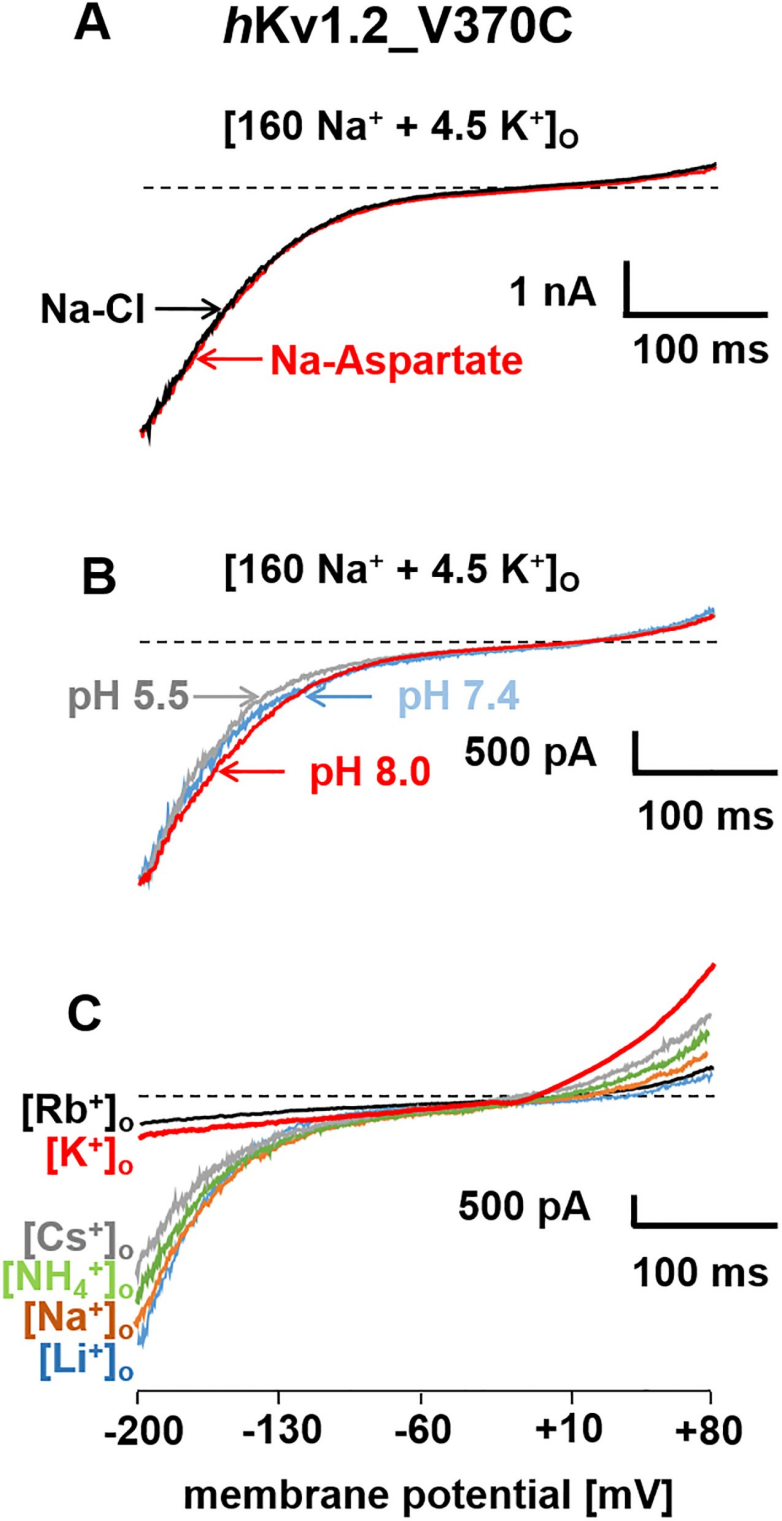
Ion conduction in the *h*Kv1.2_V370C mutant channel. Ramp currents through *h*Kv1.2_V370C mutant channels were elicited as described in the legend to Fig 1 in different external bathing solutions. The main anions (*A*) and cations (*C*) in the bathing solution or the pH of the external bathing solution (*B*) are shown at each current trace.

To further characterize the inward current in the *h*Kv1.2_V370C mutant channel we determined which ions could pass through the σ-pore. Replacing extracellular Cl^-^ by aspartate as shown in Fig 4*A* did not change the inward current suggesting that the inward current was not selective for Cl^-^.

Is the inward current through *h*Kv1.2_V370C mutant channels insensitive to protons similar to the situation in the *h*Kv1.3_V388C mutant channel [8]? To answer this question we tested external bathing solutions [160 Na^+^ + 4.5 K^+^]_O_ with different pH_O_. Decreasing pH to 5.5 or increasing pH to 8.0 did not influence σ-current (blue and red traces, Fig 4*B*) through the *h*Kv1.2 V370C mutant channels similar to what was described for current through the *h*Kv1.3_V388C mutant channel [8]. In both cases, the σ-current was not carried by H^+^.

To elucidate which ions could generate σ-currents we replaced the major cations in the external bathing solution. Extracellular Rb^+^ and K^+^ generated very small inward currents through *h*Kv1.2_V370C mutant channels whereas extracellular Cs^+^, NH_4_^+^, Na^+^ or Li^+^ could carry larger inward currents at potentials more negative than -100 mV. From the amplitudes of the ramp currents (*I*_*x*_^*+*^) at -180 mV we calculated the ratios (*I*_*x*_^*+*^*/I*_*Na*_^*+*^) as measure of ion conductance. The measurement resulted in an ion permeation efficiency in the following order: Li^+^ (1.1) >Na^+^ (1) >NH_4_^+^ (0.7) >Cs^+^ (0.3) > K^+^ (0.18) >Rb^+^(0.12) similar to what was described for currents through the σ-pore of *h*Kv1.3_V388C mutant channels [8].

### Model of the σ-pore

**Fig 5:**
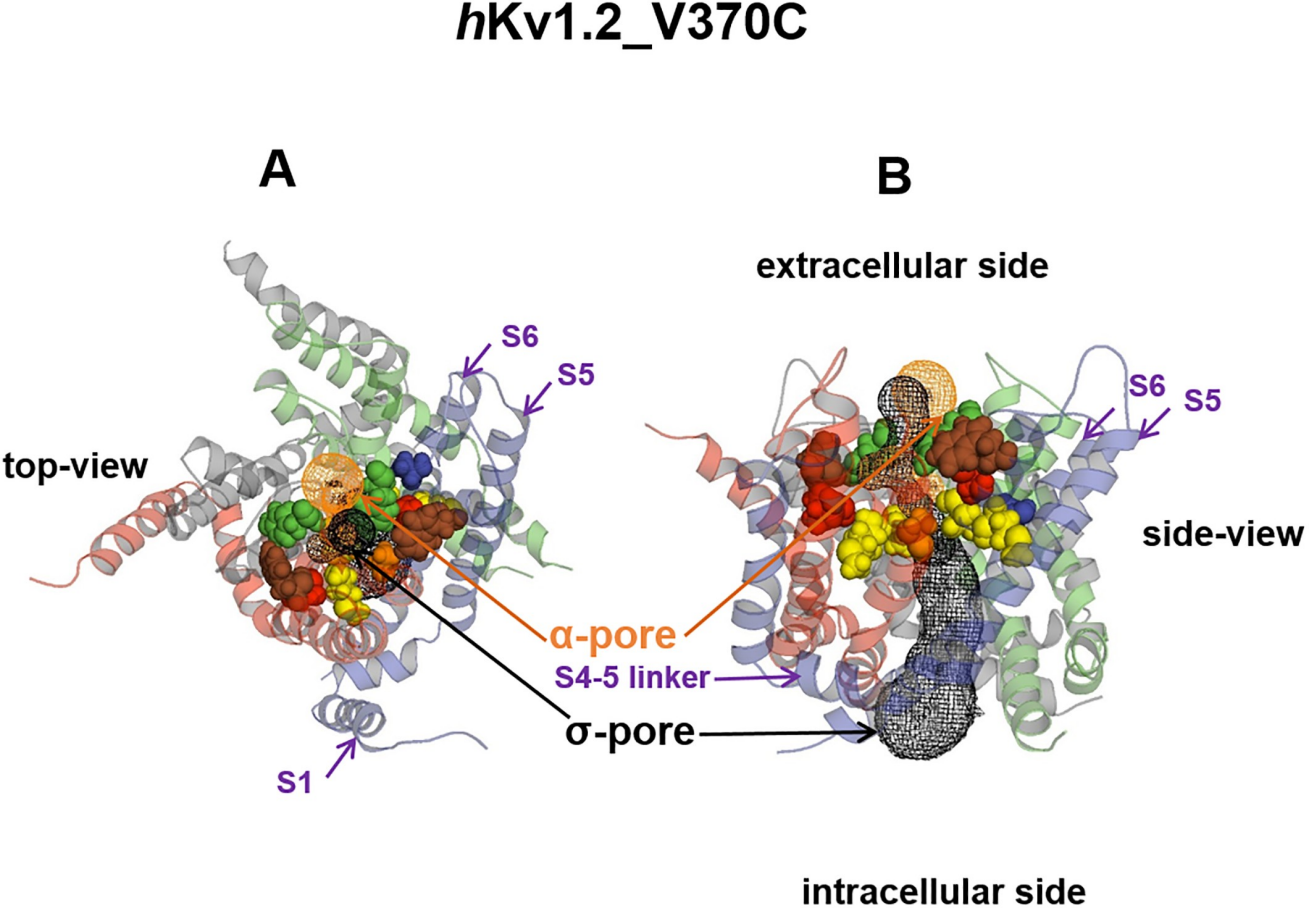
Proposed σ-pore pathway through the *h*Kv1.2_V370C mutant channel. The σ-pore is shown in black, the α-pore is shown in brown.

Prütting et al. [8] modelled the σ-pore of the *h*Kv1.3_V388C mutant channel and according to their model postulated that the entrance of the σ-pore from the outside should be located between Y395 (*Shaker* position 445) on the backside of the central α-pore and W384 (*Shaker* position 434) of the channel. Since the S5-P-S6 region of *h*Kv1.3 is very similar to *h*Kv1.2 we modelled the σ-pore in *h*Kv1.2_V370C similar to what was described for the *h*Kv1.3_V388C mutant channel [8] i.e. using the Caver program, visualizing the pore with PyMOL^®^ and verifying it with PoreWalker as shown in Fig 5. For the *h*Kv1.2_V370C mutant channel the entry of the σ-pore is located on the extracellular side of the channel between Y377 (*Shaker* position 445) on the back surface of the α-pore and W366 (*Shaker* position 434), it runs parallel to the GYG motif of the selectivity filter in the S6-S6 interface gap and ends between S5 and S6 at the intracellular side of one α-subunit.

The ending of this pathway might be responsible for the fact that σ-current can only occur in a potential range where the α-pore is closed, i.e. the voltage sensor S4 is in its resting position. The position of S4 seems to be important for the opening or closing of the σ-pore. During hyperpolarization or at the resting potential of a cell, the gap between S5 and S6 is larger (see Fig 7 of [11]). During depolarizations of the channel the voltage sensors S4 move towards the extracellular side leading to a concerted movement of S5 and S6 via the S4-S5 linker. This results in a structural change of the channel narrowing the gap between S5 and S6 [11–13]. The gap between S5 and S6 could then be too narrow to allow the flux of Na^+^ through the σ-pore. Therefore to open the σ-pore the voltage sensor S4 must be in its resting position. One could speculate that S4 moves even further towards the intracellular side or even tilts towards the side at strong hyperpolarized potentials to widen the σ-pore thereby increasing current amplitude towards more negative potentials. Such a movement would be slow (>30 ms) as can be judged by the time course of activation of the σ-current compared to the classical gating charge movements observed when opening or closing the α-pore (<3 ms).

## Conclusion

The newly described permeation pathway of the mutant *h*Kv1.2_V370C channel is likely to be similar to the σ-pore described in *h*Kv1.3_V388C mutant channels [8]. In both channels, α-pore blockers were unable to block current through the σ-pore. In addition, σ-pore current had a similar potential range of activation (more negative than -100 mV) and had the same ion selectivity. We conclude that the V370C mutation in *h*Kv1.2_V370C channels opens up a similar pathway like in the *h*Kv1.3_V388C mutant channel suggesting that the observation of a σ-pore is not restricted to Kv1.3 channels but may be a common structural element of a variety of voltage-gated ion channels. Therefore this finding could have implications for the interpretation of the cause and the treatment of different ion channel diseases associated with mutations in the pore-region of the respective channels reviewed in [14]. In such a scenario the observation of Na^+^ currents leading to long depolarizations resulting in arrhythmias [15–16] or migraine [17] could be interpreted as a result of a current similar to the σ-pore current and treatment would then require the development of a selective σ-pore blocker.
